# Quantitative assessment of the CD26+ leukemic stem cell compartment in chronic myeloid leukemia: patient-subgroups, prognostic impact, and technical aspects

**DOI:** 10.18632/oncotarget.9108

**Published:** 2016-04-29

**Authors:** Martin Culen, Marek Borsky, Veronika Nemethova, Filip Razga, Jiri Smejkal, Tomas Jurcek, Dana Dvorakova, Daniela Zackova, Barbora Weinbergerova, Lukas Semerad, Irina Sadovnik, Gregor Eisenwort, Harald Herrmann, Peter Valent, Jiri Mayer, Zdenek Racil

**Affiliations:** ^1^ Department of Internal Medicine, Hematology and Oncology, Faculty of Medicine, Masaryk University, Brno, Czech Republic; ^2^ Department of Internal Medicine, Hematology and Oncology, University Hospital Brno, Brno, Czech Republic; ^3^ Polymer Institute of the Slovak Academy of Sciences, Bratislava, Slovak Republic; ^4^ Department of Internal Medicine I, Division of Haematology and Hemostaseology, Medical University of Vienna, Vienna, Austria; ^5^ Ludwig Boltzmann Cluster Oncology, Medical University of Vienna, Vienna, Austria; ^6^ Central European Institute of Technology (CEITEC), Masaryk University, Brno, Czech Republic

**Keywords:** CML, LSC, DPPIV/CD26, FISH, FACS

## Abstract

Little is known about the function and phenotype of leukemic stem cells (LSCs) in chronic myeloid leukemia (CML) or about specific markers that discriminate LSCs from normal hematopoietic stem cells (HSCs). CD26 has recently been described as a specific marker of CML LSCs. In the current study, we investigated this marker in a cohort of 31 unselected CML patients. *BCR/ABL1* positivity was analyzed in highly enriched stem cell fractions using fluorescence *in situ* hybridization (FISH) and reverse transcription PCR (RT-PCR). The proportion of CD26^+^ LSCs and CD26^−^ HSCs varied considerably among the patients analyzed, and the percentage of CD26^+^ cells correlated with leukocyte count. The CD26 expression robustly discriminated LSCs from HSCs. This required a strict gating of the stem cell compartment. Thus, in patients with very low LSC or HSC numbers, only the highly sensitive RT-PCR method discriminated between clonal and non-clonal cells, while a robust FISH analysis required larger numbers of cells in both compartments. Finally, our data show that the numbers of CD26^+^ CML LSCs correlate with responses to treatment with BCR-ABL1 inhibitors.

## INTRODUCTION

The *BCR-ABL1* oncogene is a driver of initiation and progression in chronic myeloid leukemia (CML) [1]. The tyrosine kinase inhibitors (TKIs) directed against the BCR-ABL1 oncoprotein have proven to be successful in the treatment of CML. Today, CML patients benefit from long-term responses induced by imatinib and other *BCR-ABL1* TKIs [2, 3]. However, leukemic stem cells (LSCs) often survive TKI therapy and may be responsible for treatment failure and relapse [4, 5]. The LSC resistance to TKIs can result from acquired mechanisms, such as the selection of subclones with mutations in the *BCR-ABL1* oncogene, or may involve intrinsic mechanisms, such as LSC dormancy [6–8]. Moreover, increasing evidence suggests an important role of the microenvironment in LSC resistance [3, 9].

Current research in CML has focused on the identification and characterization of LSCs. This might enable eradication of LSCs and provide a curative therapy in CML. However, the identification of LSCs and their separation from normal hematopoietic stem cells (HSCs) in CML is challenging, since both populations reside in the same compartment phenotypically defined as CD45^+^34^+^38^−^ [10]. Recently, several groups have reported on more or less specific LSC markers and LSC-related light scatter properties in CML [10–16]. One of such markers appears to be IL-1RAP, while another is CD26, which is also known as dipeptidylpeptidase IV (DPPIV). This functionally relevant cell surface antigen as well as IL-1RAP is specifically expressed on CML LSCs, but not on HSCs [10]. LSC-specific markers, such as IL-1RAP or CD26, may also represent suitable targets for anti-LSC therapy as well as potential prognostic markers [17]. More recent data suggest that the levels of CD26 on CML LSCs may vary from patient to patient [10, 17]. The aim of this study was to investigate whether CD26^+^ LSC and CD26^−^ HSC populations can be identified and discriminated from each other in an unselected cohort of patients with *de novo* chronic phase (CP) CML. Specifically, we determined whether these two stem cell (SC) populations exclusively contain clonal or non-clonal cells using fluorescence *in situ* hybridization (FISH) and reverse transcription PCR (RT-PCR) analysis. Furthermore, we compared the light-scatter properties of CD26^+^ and CD26^−^ SCs. Finally, we asked whether the numbers of CD26^+^ CML LSCs correlate with treatment responses in CML patients of this study.

## RESULTS

### CML patients can be divided into three groups based on the percentage of CD26^+^ SCs

**Figure 1:**
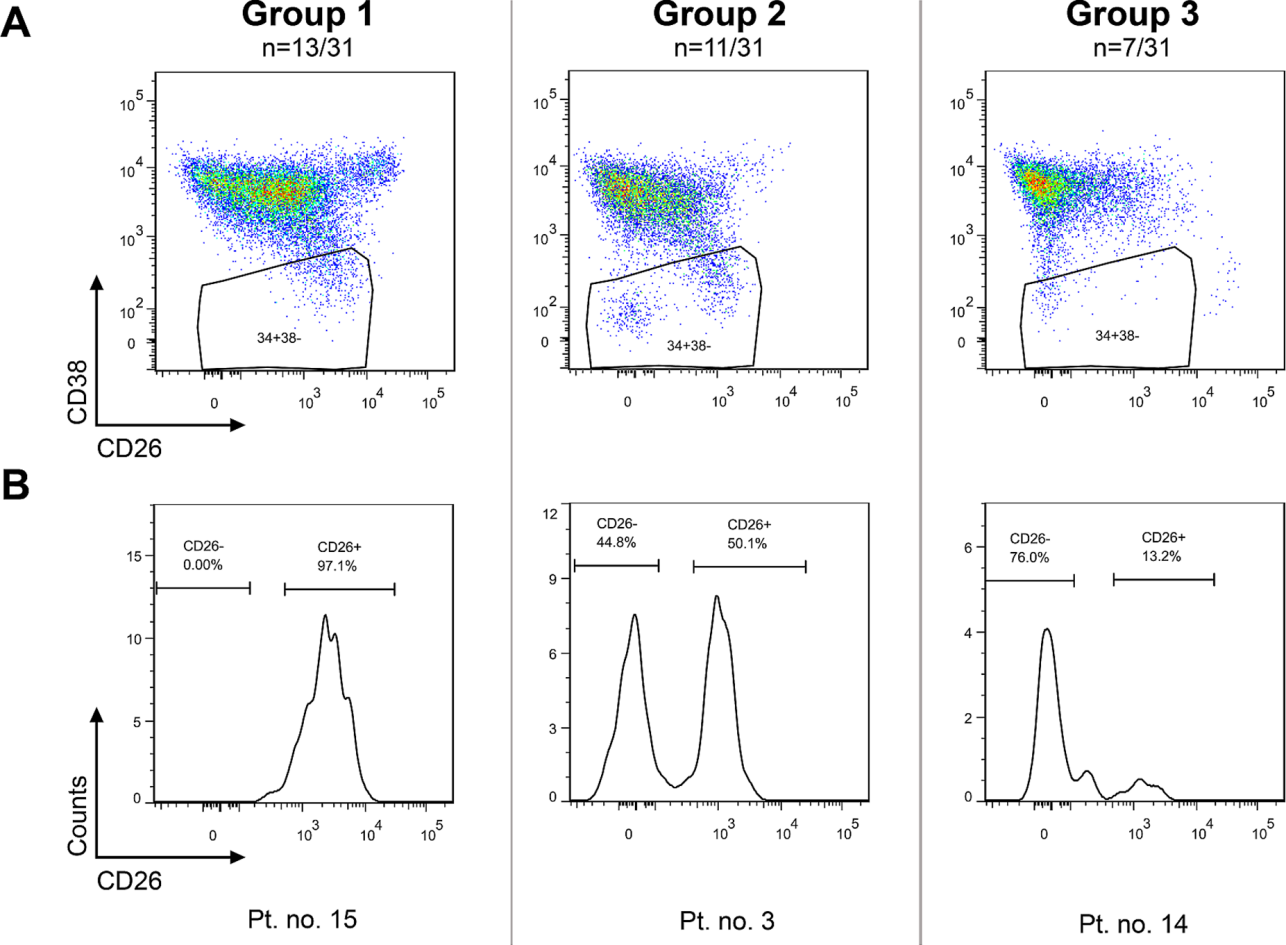
CP CML patients can be categorized into 3 groups based on CD26 expression pattern on CD45^+^34^+^38^−^ SCs Group 1: dominant CD26^+^ SC population; Group 2 – comparable numbers (percentages) of CD26^+^ and CD26^−^ SC populations; Group 3 – minor population of CD26^+^ SCs. The results for one representative patient per group are shown as dot plots (upper series) **(A)** and corresponding histograms (lower series) **(B)**. Pt. no. – patient number.

**Figure 2:**
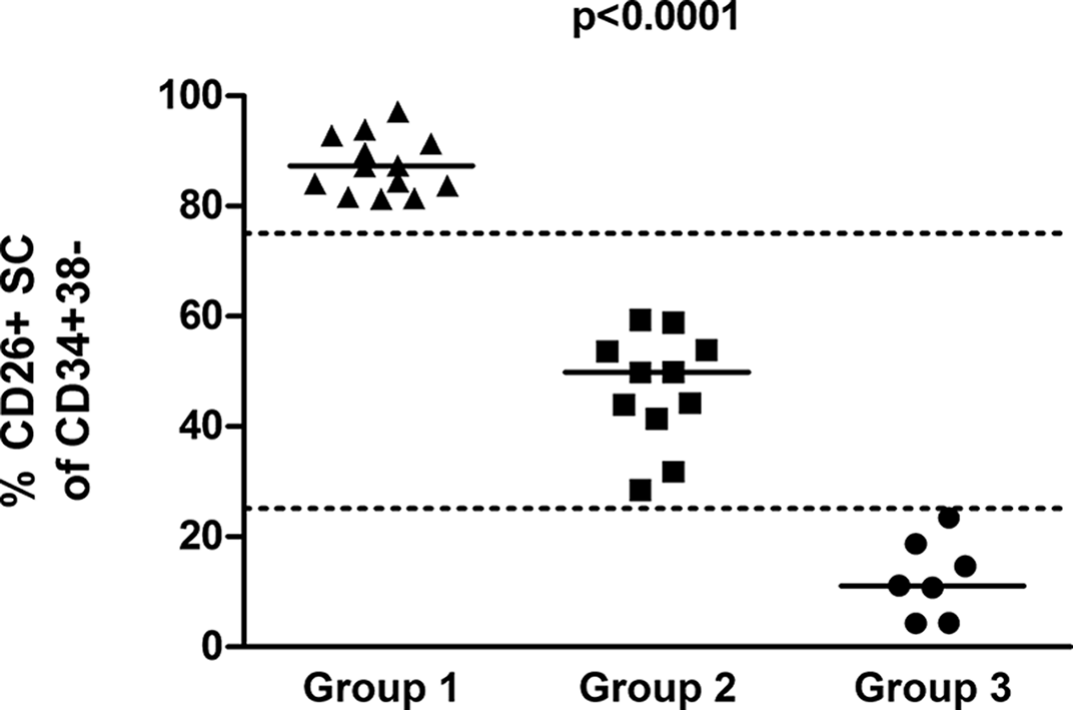
Percentage of CD26^+^ cells in the CD45^+^34^+^38^−^ SC population for the 3 patient groups, as determined by flow cytometry (*p* < 0.0001; Kruskal-Wallis; ANOVA)

The CD26 expression on CD45^+^34^+^38^−^ cells was analyzed using flow cytometry in bone marrow samples of 31 patients. The CD45^+^34^+^38^−^ compartment represents a stem cell-enriched fraction which is known to contain the most primitive blood cells, comprising true stem cells as well as multipotent progenitor cells [18, 19]. In this article, the CD45^+^34^+^38^−^ compartment is simply referred to as the “stem cells (SCs)”. The investigated CD26^+^ and CD26^−^ SC populations were well identifiable, although they varied in size among patients and were sometimes very small or even missing in some sets of the patients (Figures 1–2). Overall, three patterns of expression of CD26 on SCs were observed and the patients were categorized into 3 groups accordingly: Group 1 was characterized by a dominant CD26^+^ SC population, Group 2 by similar ratio of CD26^+^ and CD26^−^ SCs, and Group 3 by a dominant CD26^−^ SC compartment (Table 1).

**Table 1:** Delineation of 3 CML patient groups based on the percentage of CD26^+^ SCs

Group	Patient numbers	% CD26^+^ cells of CD45^+^ 34^+^ 38^−^SCs	Proportion of SC populations
1	13/31 (42%)	75–100	dominant CD26^+^
2	11/31 (36%)	25–75	similar ratio of CD26^+^ and CD26^−^
3	7/31 (22%)	0–25	dominant CD26^−^

*n* – number of patients, Pt. no. – patient number.

### FISH analysis suggests the presence of LSCs in various SC compartments

We initially applied FISH analysis in order to confirm the clonal origin of CD26^+^ SC population. A fraction of at least 1000 cells obtained by fluorescence-activated cell sorting (FACS) was required to analyze approximately 100 cells by FISH. Due to the general rarity of CD45^+^34^+^38^−^ SCs [median: 1.8 SCs per 10^4^ of all analyzed cells; interquartile range (IQR): 1.0–2.9, *n* = 27], it was not possible to obtain the required cell numbers for FISH analysis in cases with very small populations of CD26^+^ or CD26^−^ SC (Group 3 and 1, respectively). Thus, to analyze most of our patients, we applied broader gates that included the strict CD45^+^34^+^38^−^ SCs [defined by fluorescence minus one (FMO) control] and also a portion of CD38^dim^ cells. This yielded CD45^+^34^+^38^−/dim^26^+^ and CD45^+^34^+^38^−/dim^26^−^ cell fractions, further addressed as “broader CD26^+^ fraction” and “broader CD26^−^ fraction”. The CD38^dim^ region was expected to carry a mixture of LSCs, normal HSCs as well as more mature precursor cells (CD34^+^ progenitor cells). This approach allowed us to perform FISH analysis in 26/31 patients. In 5/31 patients, we were unable to isolate enough cells for FISH analysis.

**Figure 3:**
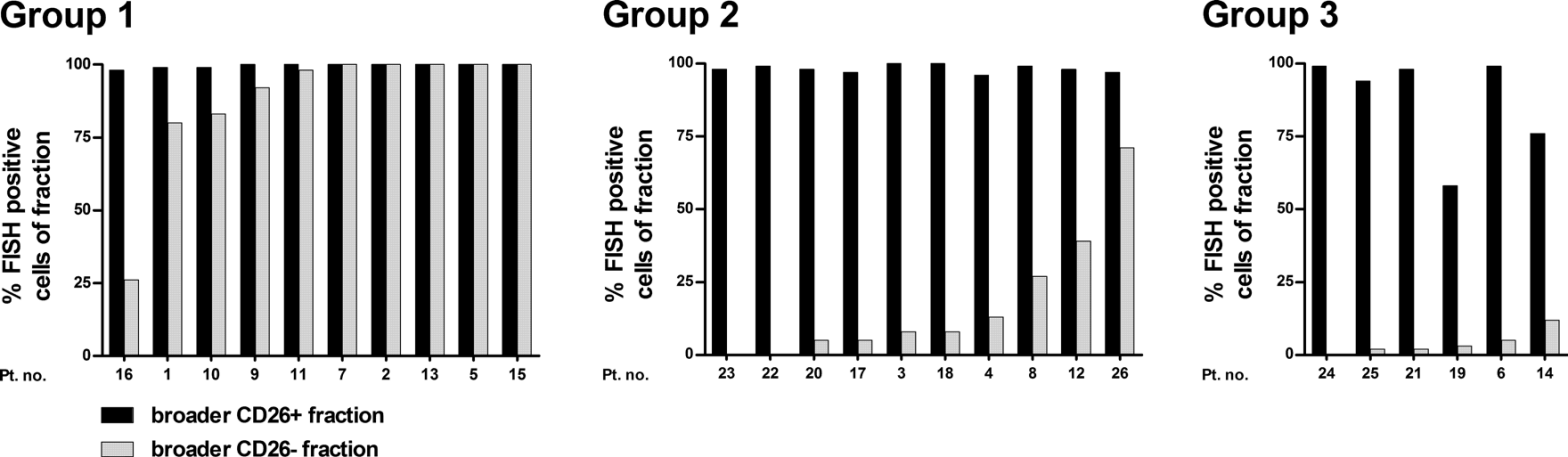
*BCR-ABL1* positivity assessed by FISH in FACS-purified broader (CD45^+^34^+^38^−/dim^) CD26^+^ and CD26^−^ fractions; shown separately for Group 1, 2, and 3. Pt. no. – patient number

In all 26 analyzed patients, almost all cells in the broader CD26^+^ fractions were *BCR-ABL1*+ by FISH (median: 99.0%; IQR: 97.8–100.0) (Figure 3). As expected, we also detected *BCR-ABL1*+ cells in the broader CD26^−^ fractions. Here, the median percentage of *BCR-ABL1*+ cells was low in the patients who carried a considerable CD26- SC population, i.e. Group 2 and 3 – 8.0% (IQR: 3.8–30.0%) and 2.5% (IQR: 1.5–6.8%), respectively. No *BCR-ABL1*+ cells were detected in the CD26^−^ fraction of only three patients from Group 2 and 3. In contrast, in Group 1, where almost no CD26^−^ SCs were previously detected and a significant contamination of CD38^dim^ (clonal) cells was expected, most of the cells in the broader CD26^−^ fraction were *BCR-ABL1*+ (median: 99.0%; IQR: 82.3–100.0). For control purposes, we also analyzed the CD45^+^34^+^38^+^ cells (further addressed as “purified progenitor fraction”) in 29/31 patients and found that this fraction contained high numbers (percentages) of *BCR-ABL1*+ cells (median: 99.0%; IQR: 95.0–100.0).

### RT-PCR analysis confirms that CD26 staining can safely discriminate between LSCs and HSCs in all three groups of CML patients

**Figure 4:**
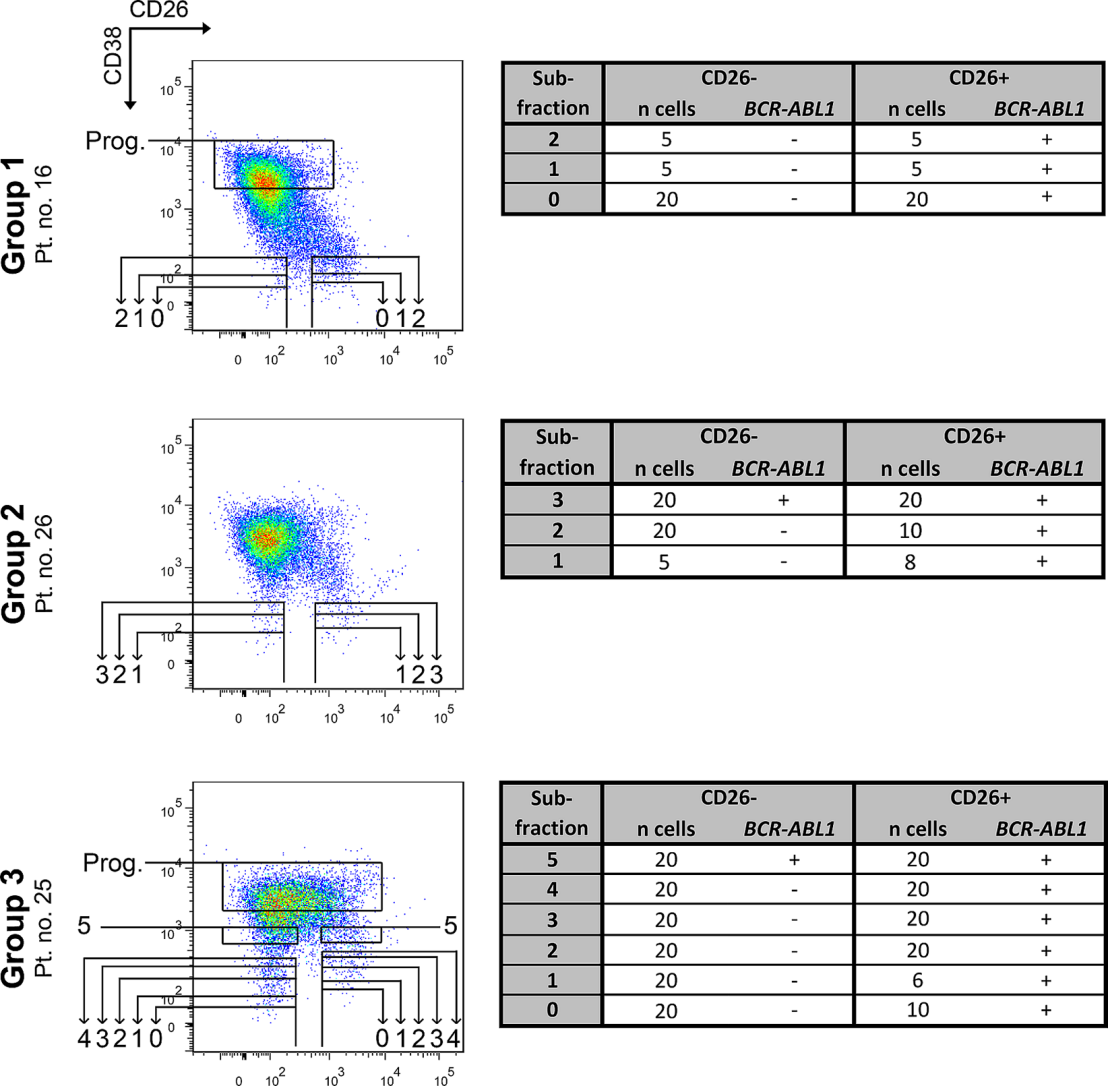
RT-PCR analysis of FACS-purified subfractions from the CD45^+^34^+^ cell compartment The subfractions numbered 0–4 contain all cells below the corresponding horizontal line. Thus, each superior subfraction also nests all previous subfractions and provides their partial repetition. Subfraction no. 5 and progenitor subfraction contain only cells from the indicated box. Subfraction no. 1 represents CD45^+^ 34^+^ 38^−^ SCs gated according to fluorescence minus one control for the CD38 antigen. Subfraction no. 0 represents even stricter gating and was isolated only if possible. Individual subfractions 0–5 were sorted and analyzed 1–2×, always with the same cell number and identical results from both analyses. Tables show the number of FACS-purified cells per one analysis and the *BCR-ABL1* status of each subfraction: (−) – negative, (+) – positive, n – number of, Prog. – more mature progenitor cells, Pt. no. – patient number.

In 3 patients analyzed by FISH (1 patient per group), we also performed a more detailed assessment of the distribution of leukemic versus normal cells in the whole CD45^+^34^+^ compartment using an RT-PCR analysis. We focused on verifying the *BCR-ABL1* negativity of CD26^−^ SCs, which could not be fully proven by FISH. In these experiments, CD26^−^ and CD26^+^ cells of the CD45^+^34^+^ compartment were FACS-purified into 3–5 subfractions (Figure 4) based on their increasing CD38 expression. Each subfraction contained variable numbers of cells ranging from 5 to 20 per one well/reaction. Using this method we were able to a) analyze even very strictly gated populations of CD38^−^ SCs (matching FMO control for CD38), without any possible contamination of CD38^+/dim^ cells; b) map the CD38^dim^ region for the distribution of *BCR/ABL1*+ cells. The method was capable of detecting a positive signal from as little as one cell, as demonstrated in positive control wells (see Methods section).

All of the CD26^+^ subfractions were proven to be *BCR-ABL1*+ irrespective of CD38 expression. In contrast, the CD26^−^ subfractions which corresponded to strictly CD38^−^ gated cells contained only *BCR-ABL1* negative cells, and the positivity started to “occur” only in subfractions with CD38^dim^ expression, which supposedly contained the leukemic CD34^+^ progenitor cells (subfractions no. 3–5; Figure 4).

### CD26^−^ SCs show low forward scatter (FSC)

**Figure 5:**
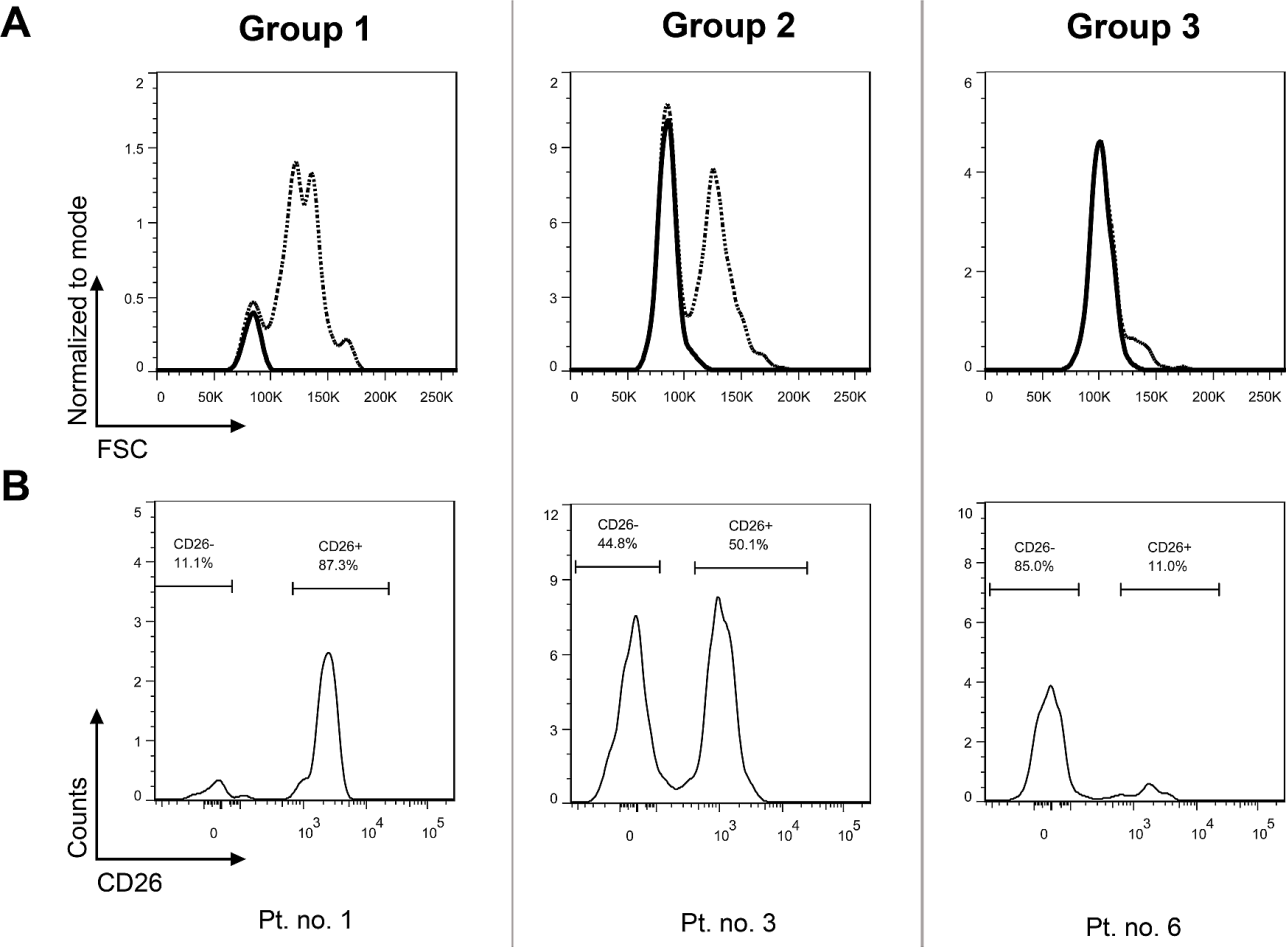
CD26^−^ SC population in CP CML is defined by low FSC Upper row shows FSC histograms (dashed line) for the entire SC population (CD45^+^ 34^+^ 38^−^), with the CD26^−^ SC population highlighted as a solid line **(A)**. The CD26^−^ SC populations were gated from CD26 SC expression histograms in the lower row of identical patients **(B)**. Pt. no. – patient number.

In order to verify alternative options for identifying and separating LSCs and HSCs, we performed additional visualization of the CD26^−^ SC population in FSC histograms (Figure 5). The FSC^low^ population perfectly matched the CD26^−^ SC population, which was nicely demonstrated in Group 2 and 3 patients. In Group 1 patients who virtually lacked CD26^−^ SCs, the FSC^low^ population was also absent (Figure 5).

### Correlation of the percentage of CD26^+^ SCs with clinical parameters in CML patients

We further assessed whether the percentage of CD26^+^ SCs and the resulting group categorization correlated with clinical or prognostic parameters in our CML patients (Table 2). A statistically significant difference was found between the mean white blood counts (WBC) at the time of diagnosis among the 3 groups (*n* = 31, *p* < 0.001; Kruskal-Wallis; ANOVA). In particular, as expected, patients with higher WBC were found to have higher levels (percentages) of CD26^+^ SCs within the total SC compartment. We also correlated response to imatinib with the percentage of CD26^+^ SCs in a homogeneous subgroup of first-line imatinib-treated patients (*n* = 15). In these patients, achievement of major molecular response (MMR) at month 12 (*n* = 15) was significantly different among the 3 groups, with the best response seen in Group 3 patients (*p* < 0.05; Fisher's exact test). However, no such relationship was found at month 18, when 12/13 patients had already achieved MMR (*p* = 0.54; Fisher's exact test; 2/15 patients were not analyzed at this time point). None of the 15 patients were switched from imatinib during these follow-up periods. No relationship was found between the patient groups defined by percentages of CD26^+^ SCs and risk stratification according to Hasford (*n* = 23, *p* > 0.05; Fisher's exact test), Sokal (*n* = 24, *p* > 0.05; Fisher's exact test), or EUTOS (*n* = 24, *p* > 0.05; Chi-square test) score. Also, no statistically significant difference was found between the frequency of either all [Common Terminology Criteria for Adverse Events (CTCAE) grade 0–5] or severe (CTCAE grade 3–5) hematological toxicities developed during the first year of TKI therapy among the 3 patient groups (*n* = 25, *p* > 0.1; Fisher's exact test).

**Table 2:** Differences in clinical and prognostic data among the 3 patient groups

	Group 1	Group 2	Group 3
**WBC** (mean ± SD, 10^9^/L;*p* < 0.001)	230.7 ± 142.5	105.6 ± 72.1	27.9 ± 9.7
**Hasford score** (n patients per Low/Int/Hi risk group; *p* > 0.05)	4/5/2	3/3/1	0/5/0
**Sokal score** (n patients per Low/Int/Hi risk group; *p* > 0.05)	3/5/3	4/3/1	1/4/0
**EUTOS score** (n patients per Low/Hi risk group;*p* > 0.05)	8/3	6/2	3/2
**Achievement of MMR on imatinib at M12** (*n* patients; *p* < 0.05)	0/3 (0%)	3/6 (50%)	6/6 (100%)
**Achievement of MMR on imatinib at M18** (*n* patients; *p* > 0.05)	3/3 (100%)	3/4 (75%)	6/6 (100%)

Hi – high; Int – intermediate; M – month; n – number.

## DISCUSSION

Recently, Herrmann et al. analyzed various surface antigens that were formerly hypothesized to define CML LSCs [17]. CD26 was reported to provide the highest specificity, which was confirmed by a series of thorough experiments including long-term culture-initiating cell (LTC-IC) and xenotransplantation assays. In the current study, we complemented these results by demonstrating the general applicability of this concept in CP CML patients. We also defined 3 patient groups based on different percentage of CD26^+^ cells in the stem cell-enriched compartment.

Clear identification of the CD26^+^ and CD26^−^ SC populations was possible in each patient, even when one of the populations was almost missing. A quantitative analysis of CD26^+^ SCs enabled us to categorize our CML patients into 3 groups. A similar patient distribution was formerly proposed by Janssen et al., who used light scatter properties to define the LSCs and HSCs [15]. In their study, 15/40 (37.5%) patients were found to carry only an LSC population (defined by higher CD34/CD45 expression and higher FSC/SSC characteristics), which corresponds to our Group 1 (42%); 25/40 patients had a varying ratio of both LSC and HSC populations (cells with lower CD34/CD45 expression, but defined by higher or lower FSC/SSC characteristics, respectively), which corresponds to our Group 2 and 3. Similarly, we found that the FSC- and CD26-based identification of HSCs matched. Although, the CD26 expression alone perfectly distinguished LSCs from HSCs, as demonstrated using the RT-PCR analysis, we propose that the combination with FSC might offer improved resolution. This might be important in problematic cases or during initial optimization experiments. Nevertheless, caution must be taken, since various red cell lysis reagents affect light scatter properties differently.

In order to determine the clonality of the stem-cell enriched fractions, we applied a robust FISH analysis and a broader gating strategy. This enabled us to analyze most of our patients, but resulted in a contamination of the CD26^+^ and CD26^−^ SC compartments with clonal cells from the CD38^dim^ region. This produced positive signals in the broader CD26^−^ fractions, most notably in Group 1 patients who only carried minor CD26^−^ SC populations. The existence of clonal cells in regions with higher CD38 expression was also supported by the fact that almost all CD38^+^ cells among all patients were found to be clonal. In summary, the FISH analysis confirmed that the CD26^+^ fraction contains almost exclusively clonal cells in all patients analyzed and that the CD26^−^ fraction represents predominantly normal HSCs in Group 2 and 3 (without a dominant CD26^+^ LSC fraction). However, the method did not prove CD26 expression to be a fully discriminatory marker and was unsuitable for analysis of Group 1 patients with minor CD26^−^ SC fractions.

In order to analyze small, strictly gated fractions, and clearly prove the discriminatory value of CD26, we analyzed one representative patient from each group using a sensitive RT-PCR method. While the analyzed patients showed FISH positivity in the broader CD26^−^ fraction, no leukemic cells were found by RT-PCR in CD26^−^ subfractions with strict CD38^−^ expression. The RT-PCR analysis thus proved that CD26 is a specific marker for CML LSCs and confirmed a crucial role of the proper CD38^−^ SC gating. In patient no. 26, the method showed that *BCR/ABL1*+ cells might already occur in the CD38^dim^ region close to the CD38^−^ gate. This particular patient showed high *BCR/ABL1*+ contamination in FISH analysis when using the broader gating strategy, which demonstrates that inaccurate CD38^−^ gating is likely to result in contamination by leukemic CD34^+^ progenitor cells from the CD38^dim^ region. This pitfall might be easily prevented by adhering to the FMO control for CD38 when modifying or setting-up a new protocol. The position of CD38^−^ gate should not change for consistently processed and analyzed samples.

The proposed patient categorization raises the question if it reflects the actual clinical status or if it has a predictive value. We suggest that a larger LSC pool would produce larger quantities of clonal descendant cells. In line with this, we showed that WBC significantly differed among our 3 groups, with Group 1 patients having the highest WBC counts and the most CD26^+^ LSCs, which also corresponds with a previous report [10]. When looking at treatment response, we found a statistically significant difference among the 3 groups in achievement of MMR at month 12, but not month 18. Janssen et al. reported that patients without residual HSCs were less likely to achieve MMR at month 18 [15]. These results indicate a relationship between the initial number of LSCs and longer time to achieve an MMR. In regard to prognostic scores, Janssen et al. found that patients with residual HSCs showed significantly better EUTOS and EURO-scores [15]. In contrast, we found no such relationship for our 3 groups, and similarly no correlation was previously found when considering only the percentage of CD26^+^ SCs [17].

The CD26/DPPIV is a multifunctional protein expressed in many tissues and cell types. This limits the potential for LSC eradication through CD26 targeting [20]. However, inhibition of CD26 by vildagliptin reduced SCID repopulating activity of CML LSC cells [10]. Moreover, in two diabetic CML patients treated with nilotinib, *BCR/ABL1* transcript level decreased after the start of gliptin therapy [10]. The potential of combined TKI and gliptin therapy for LSC eradication deserves further investigation, as gliptins are relatively safe and well characterized drugs, already widely used in treatment of diabetes.

In this study we confirmed that CD26 staining can accurately discriminate between LSCs and HSCs in all CML CP patients, which is of great clinical and diagnostic value. Additional studies are now required to determine whether the percentage of CD26^+^ SCs and the LSC/HSC proportion is of prognostic significance regarding survival and progression-free survival. In addition, further studies will be required to explore whether LSC phenotyping can be employed as a follow-up parameter in poorly responding or relapsing patients.

## MATERIALS AND METHODS

### Patients and sample collection

Bone marrow samples were collected from treatment-*naïve*, newly diagnosed CP CML patients. The patients’ characteristics are shown in Table S1. Written informed consent was provided by all patients. The study was approved by the local ethics committee of the Medical University of Vienna or ethics committee of University Hospital Brno, in accordance with the Declaration of Helsinki.

### Immunophenotyping

Immunophenotyping and FACS-purification were performed on a FACSAria III instrument (BD Biosciences, USA) and FACSDiVa 6 software using the lyse wash method. Fresh or cryopreserved leukocytes were used. Cells were either used as unprocessed cells or as cells depleted of granulocytes by anti-CD15 magnetic-activated cell sorting (MACS). Cells were stained with the following anti-human monoclonal antibodies (mAb): CD34-PE (8G12), CD34-FITC (8G12), CD26-PE (M-A261) (BD Biosciences, USA), CD45-PE-Cy7 (HI30), CD38-FITC (HIT2), CD26-APC (BA5b) (Life Technologies, USA), and CD38-PerCP-Cy5.5 (HIT2) (eBioscience, USA). The samples were immunophenotyped by one of two mAb sets: CD26-APC, CD34-PE, CD38-FITC, CD45-PE-Cy7 or CD26-PE, CD34-FITC, CD38- PerCP-Cy5.5, CD45-PE-Cy7. No changes in the proportion of CD26^+^ and CD26^−^ SCs (within the CD45^+^34^+^38^−^ compartment) were observed for fresh versus thawed samples or with regard to the different sample processing methods used, when compared directly or upon overall comparison of differently processed/stained samples (Figure S1).

### Flow cytometry analysis

Flow cytometry analysis and quantification of CD26^+^ and CD26^−^ SCs were performed using FlowJo software (gating strategy shown in Figure S2). The position of the CD45^+^34^+^38^−^ SC gate was set according to FMO control for the CD38 antigen, i.e. a sample was stained with all other antibodies except CD38, thus revealing the fluorescence spread in this channel and the correct position for the CD38^−^ gate (Figure S2). The proportion (%) of CD26^+^ and CD26^−^ SCs was calculated from distinct populations in histograms, while non-specific CD26^dim^ cells were considered as a grey zone and were excluded from the fractions to be sorted and analyzed. Due to the exclusion of these grey-zone-cells, the percentages of clearly CD26^+^ and clearly CD26^−^ SCs do not add up to 100% and represent fractions suitable for FACS-purification. The number of CD45^+^34^+^38^−^ events acquired ranged from 45 to 469 (median = 179, *n* = 31). There was no statistically significant difference between the number of CD45^+^34^+^38^−^ events acquired among the 3 patient groups (*n* = 31, *p* = 0.61; Kruskal-Wallis; ANOVA).

### FACS sorting

Different gating strategies for FACS-purification were applied for FISH and RT-PCR.

For FISH analyses, we obtained CD45^+^34^+^38^−/dim^26^+^ and CD45^+^34^+^38^−/dim^26^−^ cell fractions (broader CD26^+^ fraction and broader CD26^−^ fraction) as well as CD45^+^34^+^38^+^ cells (purified progenitor fraction). The gating strategy was patient dependent (Figure S3). Due to the low yields of the broader CD26^+^ and CD26^−^ fractions, the purity of sorting was assessed on the purified progenitor fractions (identical sorting process) and reached a purity of 99.2 ± 0.7% [mean ± standard deviation (SD), *n* = 13].

FACS-purified subfractions containing limited cell numbers (described in Results) were analyzed by direct nested RT-PCR (see below). Wells with no sorted cells were included in each PCR run as a no template control. The purified progenitor cells served as positive controls for both the FACS process and the PCR amplification (separate wells of 1–20 cells; in total: 8 × 1 cell, 9 × 5 cells, 5 × 10 cells, 6 × 20 cells). False negativity was obtained in 2/28 of these positive control wells (7%; two separate runs)

### FISH analysis

FACS-purified cells were fixed by methanol-acetic acid solution (3:1; fixative solution). FISH analysis was performed on interphase nuclei to detect the *BCR-ABL1* fusion using the XL *BCR-ABL1* plus probe (MetaSystems, Germany). Whole volume of fixative solution (15–20 μl) with resuspended cells was spotted drop-wise onto a minimal slide area and let dry on a heater-plate at 56°C for 20 min. Hybridization with the FISH probe was performed according to the manufacturer recommendations with the following modifications: 7 μl of probe mixture was applied on slide; slides were washed in a 0.4X SSC/0.3% NP-40 solution (Abbott molecular, USA) at 73.5°C for 4 min; next slides were washed in 2X SSC/0.1% NP-40 solution (Abbott molecular, USA) at room temperature for 2 min. Fluorescence signals were evaluated using a Nikon Eclipse E80i fluorescence microscope and documented with LUCIA FISH software (Laboratory Imaging, Czech Republic).

### RT-PCR analysis

FACS-purified subfractions containing limited cell numbers (as low as one cell) were analyzed for *BCR-ABL1* positivity by direct nested reverse transcription PCR. Primers were used according to a previously published method [21]. Initially, cells were lysed using guanidine thiocyanate, followed by three quick freeze-thaw cycles at −80°C. The first-step of the nested RT-PCR was performed using the AffinityScript One-Step RT-PCR Kit (Agilent Technologies, USA) and included a single-tube reverse transcription and the first PCR round [22]. The resulting PCR product was purified with a mixture of Exo I and FastAP Thermosensitive Alkaline Phosphatase at a ratio of 1:2 (Fermentas – Thermo Fisher Scientific, USA). The purified PCR product was diluted 100× in RNase-free water. For a second amplification, the HotStar Taq DNA Polymerase Kit (Qiagen, Germany) was used. Amplification products from both PCR rounds were visualized using a QX DNA Screening Kit on a QIAxcel Advanced instrument and analyzed using QIAxcel ScreenGel software (all Qiagen, Germany).

## SUPPLEMENTARY MATERIAL FIGURES AND TABLE


