# Supplementary material for: Quantitative assessment of the CD26+ leukemic stem cell compartment in chronic myeloid leukemia: patient-subgroups, prognostic impact, and technical aspects
Source: Oncotarget. 2016 Apr 29;7(22):33016–24. doi: 10.18632/oncotarget.9108 (PMC5078071; doi:10.18632/oncotarget.9108)
Supplement: Supplementary file 1 [file oncotarget-07-33016-s001.pdf]

Quantitative assessment of the CD26+ leukemic stem cell compartment in chronic myeloid leukemia: patient-subgroups, prognostic impact, and technical aspects

Supplementary Materials

Supplementary Table S1: Patient characteristics

No	Age (yrs)	Gender (m/f)	WBC (10^9/L)	Karyotype	Patient group categorization
**1**	75	m	316	46,XY,t(9;22)	1
**2**	60	f	481	46,XX,t(9;22)	1
**3**	60	m	194	46,XY*	2
**4**	44	f	46	46,XX,t(9;22)	2
**5**	39	m	243	46,XY,t(9;22),der(22)	1
**6**	69	f	24	46,XX,t(9;22)	3
**7**	37	m	435	NA#	1
**8**	72	m	44	46,XY,t(9;22)	2
**9**	41	m	98	47,XY,+8,t(9;22)	1
**10**	63	m	22	46,XY,t(9;22)	1
**11**	33	m	330	46,XY,t(9;22)	1
**12**	29	f	75	46,XX,t(9;22)	2
**13**	39	m	250	46,XY,t(9;22)	1
**14**	78	f	15	46,XX,t(9;22)	3
**15**	68	m	34	46,XY,t(9;22)	1
**16**	35	m	290	46,XY,t(9;22;19)	1
**17**	72	f	24	46,XX,t(9;22)	2
**18**	45	f	77	46,XX,t(9;22)	2
**19**	67	m	18	47,XY,+8,t(9;22)	3
**20**	63	m	250	46,XY,t(9;22)	2
**21**	76	f	34	46,XX,t(9;22)	3
**22**	70	m	150	45,X,-Y,t(9;22)	2
**23**	68	m	40	46,XY,t(9;22)	2
**24**	66	m	38	46,XY,t(9;22)	3
**25**	72	f	40	46,XX,t(9;22)	3
**26**	53	m	124	46,XY,t(9;22)	2
**27**	58	m	45	46,XY,t(9;22)	1
**28**	65	m	137	46,XY,t(9;22)	2
**29**	60	m	27	46,XY,(t9;22)	3
**30**	66	f	277	NA#	1
**31**	62	f	181	46,XX,(t9;22)	1

No – patient number; yrs – years; m – male; f – female; WBC – White Blood Count at diagnosis.
*negative cytogenetics, ***BCR-ABL1*** fusion gene detected on chromosome 9 by FISH, PCR positivity.
#without mitosis, ***BCR-ABL1*** detected by PCR.

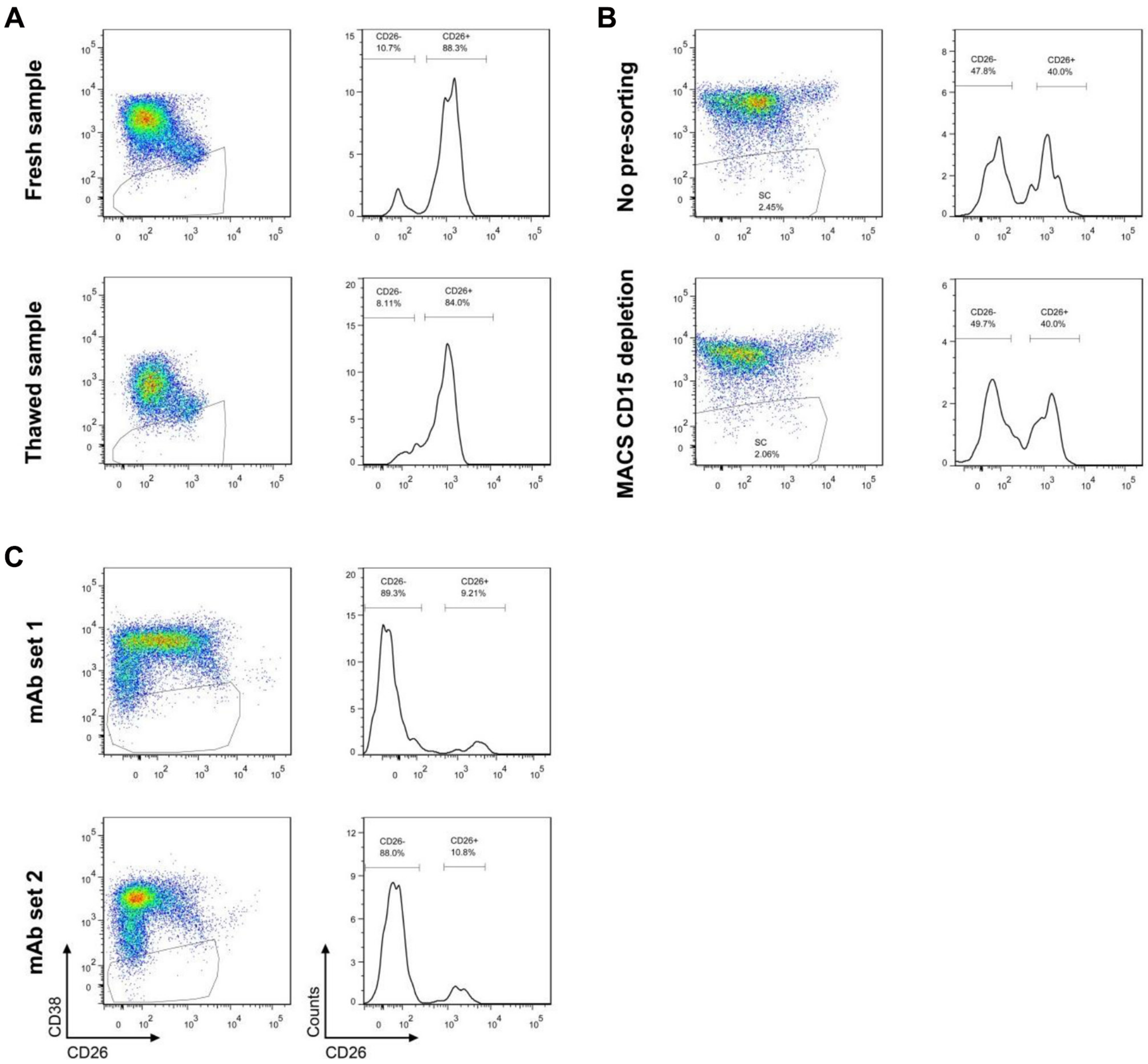

Supplementary Figure S1: Comparison of different processing of samples and two mAb staining sets used (set 1 - CD26-APC, CD34-PE, CD38-FITC, CD45-PE-Cy7; set 2 - CD26-PE, CD34-FITC, CD38-PerCP-Cy5.5, CD45-PE-Cy7). The figure shows representative examples of: a sample processed fresh and after cryopreservation (leukocytes, stained by mAb set 2) (**A**); a sample processed as leukocytes and leukocytes depleted for $CD15^{+}$ cells by magnetic-activated cell sorting (both after cryopreservation and stained with mAb set 1) (**B**); a sample stained by the two different mAb sets (cryopreserved leukocytes) (**C**).

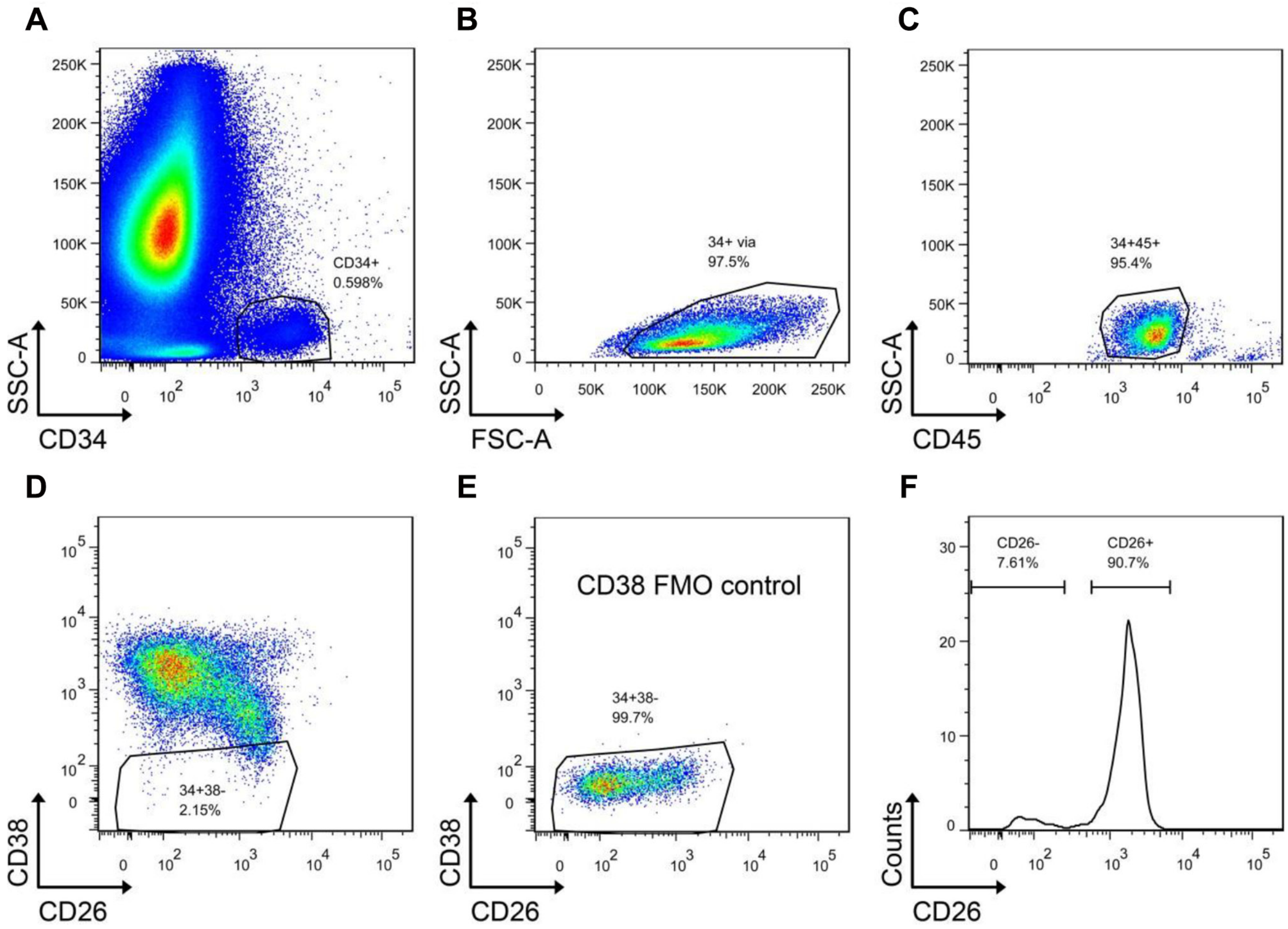

Supplementary Figure S2: Gating strategy for flow cytometry analysis and quantification (%) of CD26^{+} and CD26$^{–}$ populations from CD45^{+}34^{+}38$^{–}$ SCs. Following initial exclusion of CD45$^{–}$ cells, immature CD34^{+} cells were selected (**A**).Viable cells were then gated based on their light-scatter properties (**B**); followed by gating for CD45^{dim} blasts (**C**). The refined population was then gated for CD38$^{–}$ cells (**D**), according to a fluorescence minus one (FMO) control (**E**). The percentage of CD26^{+} and CD26$^{–}$ stem cells was calculated from histograms for distinct populations (**F**), while excluding unspecific CD26^{dim} cells.

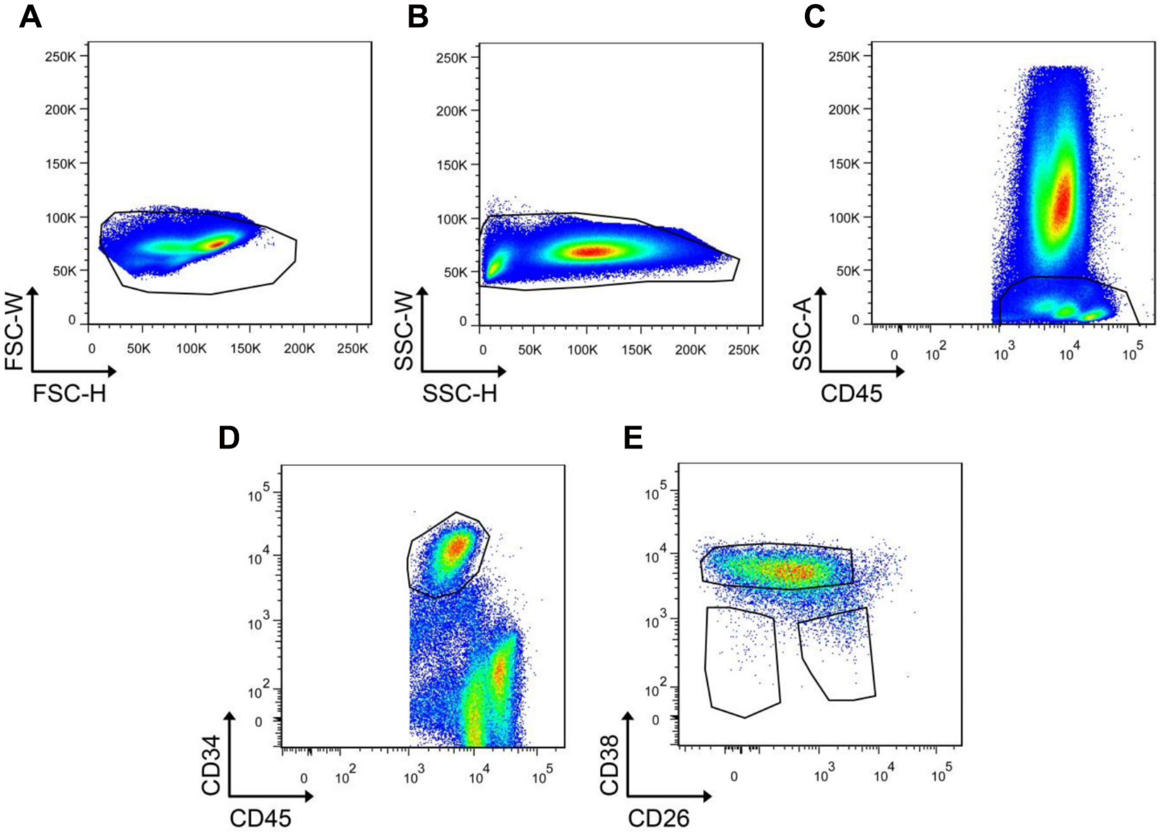

Supplementary Figure S3: Gating strategy for FACS-purification of broader (CD45$^+$34$^+$38$^{–/dim}$) CD26$^+$ and CD26$^–$ fractions. First, singlets were selected based on their light-scatter properties (**A**, **B**). Next, mononuclear leukocytes were gated (**C**), followed by gating for immature CD34$^+$ cells (**D**). Finally, the CD38$^+$ cells were purified as a progenitor fraction, and CD38$^{–/dim}$ stem cells were purified into CD26$^+$ and CD26$^–$ fraction (**E**).
